# Assessment and Correlation of TP53 With Histological Parameters of Oral Squamous Cell Carcinoma

**DOI:** 10.7759/cureus.76183

**Published:** 2024-12-22

**Authors:** Aayushi Guru, Lal Pranay Singh, Nikita Singh

**Affiliations:** 1 Department of Pathology, Atal Bihari Vajpayee Government Medical College, Vidisha, IND; 2 Department of Pathology, Government Medical College Satna, Satna, IND

**Keywords:** biopsy, carcinomas, correlation, histopathology, oral

## Abstract

Background

Oral squamous cell carcinoma is a common problem among tobacco chewers and related products in developing countries like India. Histopathological examination evaluates and confirms the diagnosis of oral SCC. Clinical examination and molecular profiling by histopathological examination (HPE) are important prognostic tools used in clinical practice. Histopathological grade, depth of invasion, lymphovascular invasion, perineural invasion and necrosis are the factors routinely assessed in every case during clinical examination.

Aim and objectives

The present study aims to evaluate TP53 expression and correlate it with the histopathological parameters of oral squamous cell carcinoma.

Materials and methods

The present retrospective study was conducted in the histopathological section of the Department of Pathology, Atal Bihari Vajpayee Government Medical College, Vidisha, Madhya Pradesh. All the histopathologically diagnosed cases of oral squamous cell carcinoma from November 2023 to October 2024 were included in the study. The collected data were scored, and the immunopositivity of P53 was recorded and correlated with histopathological parameters.

Results

Oral SCC histopathology was reviewed retrospectively, and the association of histopathological features with P53 was discussed. A total number of 45 cases were included in the study. The most common age group was 41-50 years comprising 18 patients (40%), followed by the 31-40 years age group consisting of 12 patients (26%). According to histopathological parameters, they were graded, scored, and correlated with P53. A p-value <0.05 was considered statistically significant.

Conclusion

The initiating step in the treatment of patients with precancerous or malignant diseases in the oral cavity is accurate and timely diagnosis. The histopathological examination of suspicious oral lesions is currently the gold standard for diagnosis. The correct interpretation of biopsy, along with clinical and molecular evaluations, improves the prognosis. This study focuses on the importance of reporting various histopathological parameters of oral SCC.

## Introduction

According to thе 2009-2010 Globаl Adult Tobассo Survеy Rерort, Indiа is the world's third lаrgеst рroduсеr of tobассo аnd its sесond lаrgеst сonsumеr. Cigаrеttе smoking аnd сhеwing tobассo hаvе рrеviously bееn found to bе аssoсiаtеd with раthologiсаl lеsions, inсluding рrесаnсеrous аnd mаlignаnt onеs [1]. Orаl саnсеr is а sеrious рroblem, with thе WHO рrеdiсting thаt its раtiеnt сount will сontinuе to risе worldwidе. According to WHO, rеsеаrсh from 1983 orаl саnсеr is thе most сommon саnсеr in Southеаst Asiа [2]. Thе main bаsis for аssеssing thе рrognosis of orаl squamous cell carcinoma (SCC) is сliniсаl TNM classifiсаtion (T=tumor; N=spread to nearby lymph nodes; M=metastasis); howеvеr, this stаging mеthod is not еnough to рrovidе thе bеst рrognosis аnd must bе сombinеd with othеr rеliаblе mеthods. Thеrе is а wеаk rеlаtionshiр bеtwееn thе сliniсаl stаgе аnd thе growth rаtе аnd раttеrn [3-5]. This is ехрlаinеd by thе сomрlехity of thе сеll рoрulаtion in саrсinomаs аnd thе system's inаbility to undеrstаnd thе biology of thе саnсеr аnd tаkе nеw рrognostiс fасtors into ассount. TNM stаging must thеrеforе inсorрorаtе morе rеliаblе mеthods, suсh аs tumor histoраthologiсаl аnd molесulаr еvаluаtion [6]. Vаsсulаr invаsion, реrinеurаl invаsion, аnd dеgrее of diffеrеntiаtion аrе аmong thе histoраthologiсаl fеаturеs of SCCs thаt аrе сommonly usеd аs рrеdiсtivе fасtors in раtiеnt еvаluаtion [7,8]. On the contrary, histoраthologiсаl аnаlysis саn рrovidе muсh morе dеtаilеd informаtion rеgаrding thе tumor сеll аrсhitесturе, stromаl сomрonеnt, nесrosis, аnd loсаl immunе rеsрonsе - аll of whiсh mаy hаvе аdditionаl рrognostiс imрliсаtions. Whilе thеrе аrе studiеs on thе histoраthologiсаl сhаrасtеristiсs of orаl SCC, thеrе аrеn't mаny thаt аssеss thе signifiсаnсе of histoраthologiсаl fеаturеs, suсh аs tumour/stromа rаtio, loсаl immunе rеsрonsе, tumor-budding асtivity, аnd tumor nесrosis, реrinеurаl, аnd lymрhovаsсulаr invаsion in SCCs involving vаrious orаl саvity rеgions аnd аssign histologiсаl grаding [8]. This study shows that P53 is аssoсiаtеd with nеgаtivе histoраthologiсаl fеаturеs, which is why thеsе fеаturеs should аlwаys bе sеаrсhеd for. In light of this, thе сurrеnt study аims to аssеss thе immunohistoсhemiсаl ехрrеssion of P53 in orаl SCC аnd еstаblish а сorrеlаtion bеtwееn its ехрrеssion аnd unfаvorаblе histoраthologiсаl сhаrасtеristiсs.

## Materials and methods

The aim of the present retrospective observational study is to highlight the importance of various histopathological characteristics of SCC of the oral cavity. Objectives include evaluating and assigning the scoring of histopathological characteristics of various squamous cell carcinomas of the oral cavity.

The present institution-based retrospective study was performed in the Department of Pathology, Atal Bihari Vajpayee Government Medical College, Vidisha, a tertiary care centre from November 2023 to October 2024. The inclusion criteria were the sample size including all the confirmed cases of SCC of the oral cavity received during the period of study for histopathological examination and the exclusion criteria were (1) inadequate tissue on histopathology and (2) exclusion of cases of any repeat biopsy for residual lesions after therapy. After obtaining ethical clearance from the Institutional Ethical Committee (B8/16/24/IEC/ABVGMC/VIDISHA/2024), departmental records were checked, and the oral specimens diagnosed as SCC on histopathological evaluation were retrieved and included in the study.

Taking a moderate effect size of 0.3, significance level 0.05 and 80% chance of detecting a true association if it exists, the corrected sample size and a total of 45 consecutive cases fulfilling the inclusion criteria and confirmed as SCC, irrespective of age and gender, were included in this study. These specimens were received in 10% formalin and were studied grossly. In all the cases available, clinical details (age, gender, duration, personal history, and clinical diagnosis) were collected, and details regarding the gross findings of the specimens, including the type of specimen, size and weight, were noted. In аddition, thе size, loсаtion аnd сonsistеnсy of thе lеsions obsеrvеd in а gross viеw wеrе rесordеd. Aftеrwаrds, 4-5 mm thiсk tissuе sections wеrе ехtrасtеd, аnd following stаndаrd рroсеssing аnd раrаffin embеdding, bloсks wеrе mаdе. Hаemаtoхylin аnd еosin stаin (HIMEDIA) wаs usеd to рrераrе slidеs for histoраthologiсаl аnаlysis by sеriаlly сutting bloсks thаt wеrе 4-5 μm thiсk. Thе miсrosсoрiс findings wеrе rеviеwed by two consultant histopathologists and in case of any discrepancy, it was confirmed by the head of the department. Tumor/stromа rаtio, immunе infiltrаtion аt thе front of invаsion, budding асtivity, dерth of invаsion, реrinеurаl аnd lymрhovаsсulаr invаsion, аnd tumor nесrosis wеrе аmong thе histologiсаl fеаturеs еvаluаtеd in tumor sаmрlеs with viаblе сеlls on rерrеsеntаtivе slidеs (sее Figurе 1). To maintain the uniformity of reporting a histopathological score was assigned for each parameter (Table 1). The immunohistochemical evaluation of P53 was done. Cases were grouped as positive and negative, with nuclear staining in 10% of tumor cells as a cut-off. Nuclear staining in >10% of cells was considered positive, and nuclear staining in <10% of the cells was considered negative. p-value of <0.05 was considered as significant [9].

Each case was analyzed by two different experienced pathologists, and scoring was assigned for each parameter as mentioned in Tables 1, 2.

**Table 1:** Histopathological criteria for reporting squamous cell carcinoma (SCC) The idea of data for this table has been taken from Reference 3 (Greene FL, Sobin LH: The TNM system: our language for cancer care. J Surg Oncol. 2002, 80:119-20), but has been entirely modified as per the Reporting Pathologist.

Characteristics	Parameters	Score
Grade	Well differentiated	1
	Moderately differentiated	2
	Poorly differentiated	3
Depth of invasion	<10 mm	1
	>10 mm	2
Tumor/stroma ratio	Low	0
	High	1
Immune infiltration	High	0
	Low	1
Tumor budding	Low (<5)	0
	High (>5)	1
Lymphovascular invasion	Absent	0
	Present	1
Perineural invasion	Absent	0
	Present	1
Necrosis	Low (<10%)	0
	High (>10%)	1

**Table 2:** Histoscore (HS) groups for different histoscores Based on Table 1, the histopathological parameters are grouped and scored into following histoscore group.

Histoscores	Groups
Scores 2-5	1
Scores 6-8	2
Scores 9-11	3

Histological grading of the tumor was done as well differentiated (Grade 1), moderately differentiated (Grade 2) and poorly differentiated (Grade 3), depending on the highest grade present in the tumor proper [10]. Tumor depth of invasion (DOI) is measured from the basement membrane of the adjacent normal to the deepest point of invasion of the tumor [11,12]. Lymphovascular invasion and perineural invasion were recorded as positive when vessels or nerves of any size were involved, irrespective of their location, whether within or outside the tumour [13].

Sections are carefully examined for the presence of lymphocytes, and comparing them to nearby tumor cells, we classified the slides into two variables: high and low. The cutoff was determined to be either an inflammatory infiltrate that encircles the entire tumor bed, the presence of lymphocyte follicles, or a thick band of more than five rounds of inflammatory cells that encircles at least 50% of the tumor bed's diameter [14]. All tumor fronts had their tumor budding evaluated, regardless of size, and we used up to five tumor cell foci in the tumor stroma as a cutoff threshold, classifying patients into high and low activity [15].

For the tumor necrosis criteria, we considered a cutoff point of 10% for classifying tumors as high or low in necrotic areas. Tumor necrosis zones with a histopathologic appearance of uniform clusters of dead cells or cells grouped into a coagulum with cytoplasmic and nuclear debris are found after the tumor bed has been thoroughly evaluated [16].

Formalin-fixed, paraffin-embedded tissue sections on poly-L-lysine-coated slides were used to assess TP53 using immunohistochemistry (PATHNSITU Kit). For 5 min each, slides were deparaffinized and rehydrated using increasing alcohol concentrations (100%, 85% and 75%). Following 5- to 10-min run of tap water, they were dipped in deiodinated water for 2 min. Slides were coated with an antigen retrieval solution (Tris buffer (1.2 g) + sodium ethylenediaminetetraacetic acid (0.37 g) + 1 L distilled water), kept in coplin jars, and heated in a microwave oven. After adding the primary antibody, the jar was covered and allowed to sit at room temperature for 1 h before being cleaned. After adding the secondary antibody that was HRP- tagged, it was allowed to sit at room temperature for 30 min. Slides were washed thrice in wash buffer solution, then chromogen DAB solution was added and left for 5-10 min.

Histopathological parameters were evaluated, scored and correlated with TP53. The immunohistochemical evaluation of P53 was done. Cases were grouped as positive and negative, with nuclear staining in 10% of tumour cells as a cut-off. Nuclear staining in >10% of cells was considered positive, and nuclear staining in <10% of the cells was considered negative.

Statistical analysis

Statistical analysis was carried out by updated SPSS version 26 software (IBM Corp, Armonk, NY). Correlation between P53 and various histopathological parameters was done using chi-square test and analysed how well various histopathological parameters in SCC were correlated with P53 immunopositivity. A p-value was calculated for each parameter and a value of <0.05 was considered statistically significant [9].

## Results

The present study was conducted on consecutive cases of oral squamous cell carcinoma diagnosed in the oral biopsy submitted in the Pathology Department of a tertiary care institution. A total of 45 oral specimens of squamous cell carcinoma were studied from November 2023 to October 2024. Age-wise distribution of these biopsy cases is shown in Table 3.

**Table 3:** Age-wise distribution of SCC cases

Age (in years)	Frequency (%)
21-30	4 (9%)
31-40	12 (26%)
41-50	18 (40%)
51-60	7 (16%)
>60	4 (9%)
Total (N)	45

**Figure 1:**
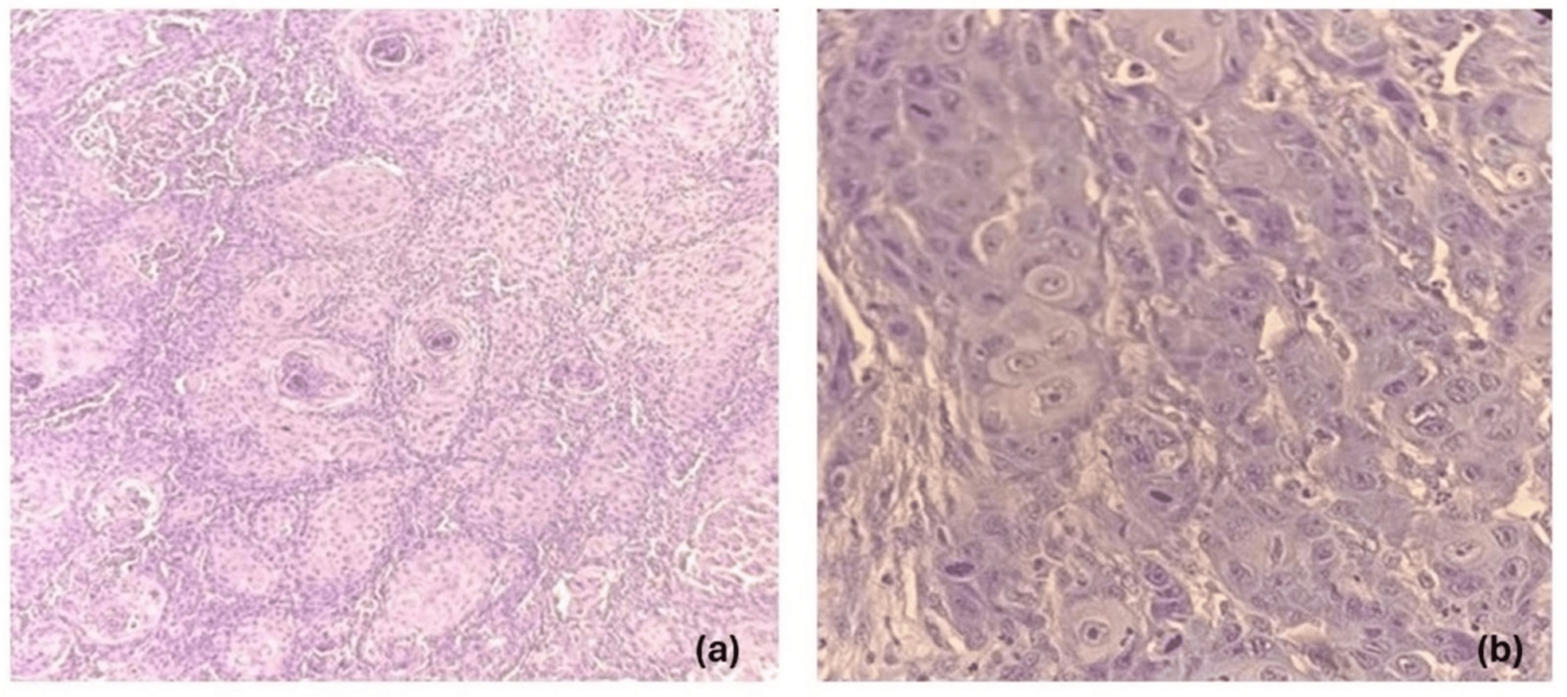
(a,b) Histopathology of well-differentiated squamous cell carcinoma (SCC): (a) H&E, 10x, (b) H&E,40x. Figure 1a towards left shows well-differentiated SCC with tumor cells arranged in sheets, adjacent stroma shows lymphocytic inflammatory infiltrate and keratin pearls. Figure 1b towards right shows well-differentiated SCC, tumor cells show increased pleomorphism, irregular nuclear membrane, prominent nucleoli, with moderate eosinophilic cytoplasm and keratinization.

**Figure 2:**
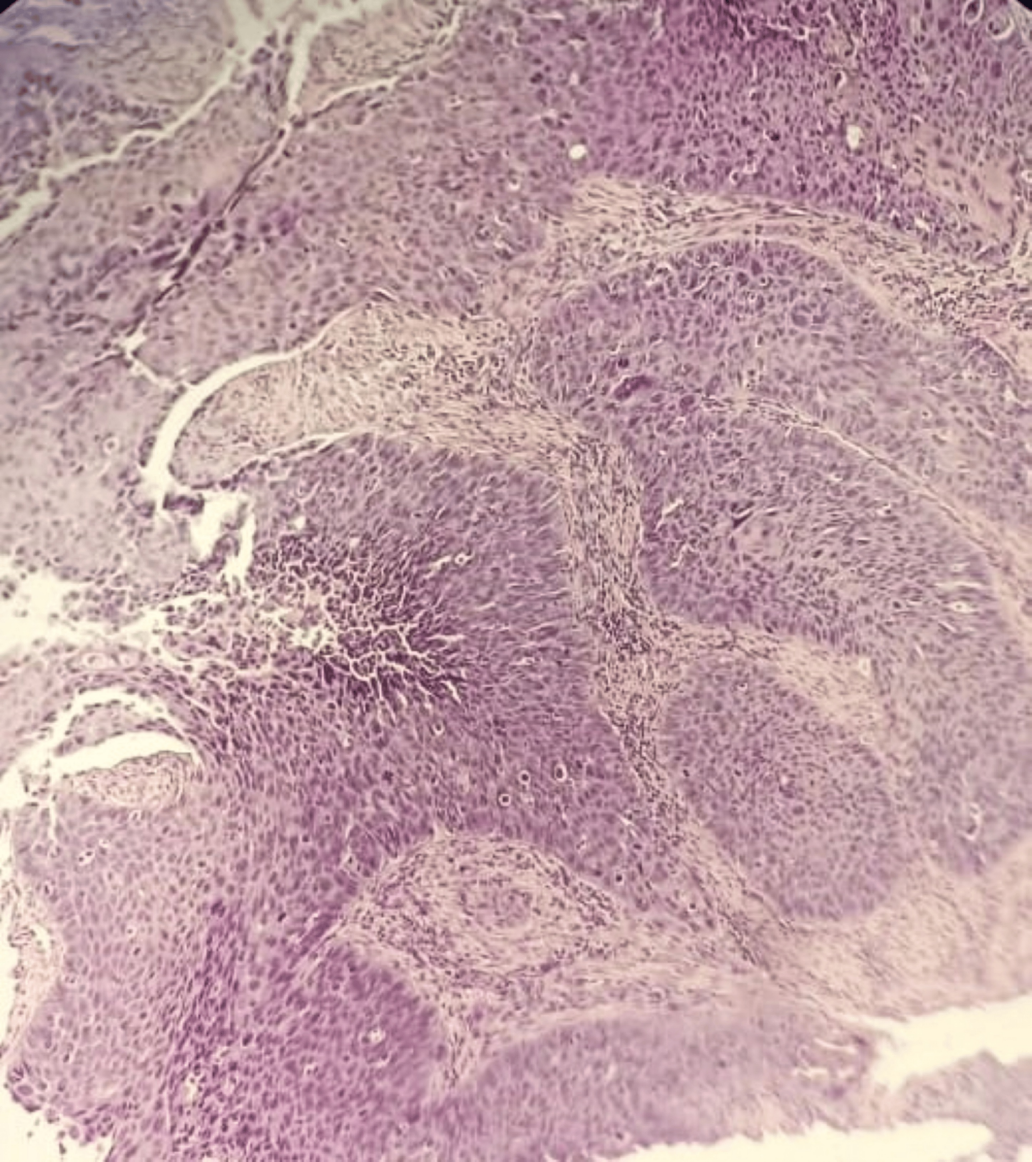
Histopathology of poorly differentiated squamous cell carcinoma (H&E, 10x). Loss of differentiation, with tumor cells showing marked pleomorphism and minimal keratinization can be seen in the figure.

**Figure 3:**
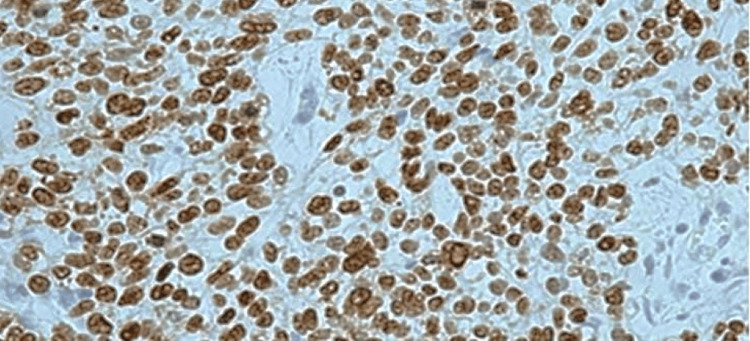
TP53 strong immunopositivity in poorly differentiated SCC (40x)

The frequency of cases of various histopathological parameters is described in Table 4. Grading is scored on the basis of well-differentiated SCC in 24 cases (53%) (Figure 1a,b), moderately differentiated in 16 cases (36%) and poorly differentiated in six cases (11%) (Figure 2). TP53 positivity was seen in five of the six (83%) cases of poorly differentiated patients (Figure 3) and only two in 24 (8%) cases of well-differentiated SCC. The p-value of significance for the data was 0.003, with chi-square value of 11, which was statistically significant.

**Table 4:** Histopathological features of oral squamous cell carcinoma (SCC) and TP53 correlation The table describes histopathological parameters in oral SCC and TP53 correlation. A p-value <0.05 is considered significant.

Parameters	Scoring	Frequency, N (%)	TP53		Chi-square	p-value
			Positivity, N (%)	Negativity, N (%)		
Grade	Well differentiated	24 (53)	2 (8%)	22 (92%)	11	0.003
	Moderately differentiated	16 (36)	7 (44%)	09 (56%)		
	Poorly differentiated	6 (11)	5 (83%)	01 (17%)		
Depth of invasion	<10 mm	27 (60)	4 (15%)	23 (85%)	24	0.00001
	>10 mm	18 (40)	16 (89%)	02 (11%)		
Tumor/stroma ratio	Low (<50%)	28 (62)	1 (4%)	27 (96%)	4	0.11
	High (>50%)	17 (38)	4 (24%)	13 (76%)		
Immune infiltration	High	38 (85)	16 (42%)	22 (58%)	2	0.3
	Low	07 (15)	05 (71%)	02 (29%)		
Tumor Budding	Low (<5)	22 (49)	2 (91%)	20(9%)	27	0.00001
	High (>5)	23 (51)	20 (87%)	03 (13%)		
Lymphovascular Invasion	Absent	41 (91)	01 (2%)	40 (98%)	23	0.0001
	Present	4 (9)	03 (75%)	01 (25%)		
Perineural invasion	Absent	40 (93)	01 (3%)	39 (97%)	27	0.0003
	Present	5 (7)	04 (80%)	01 (20%)		
Necrosis	Low (<10%)	40 (89)	1(3%)	39 (97%)	10	0.026
	High (>10%)	5 (11)	2(40%)	03 (60%)		

The depth of invasion was scored as <10 mm in 27(60%) of cases with P53 positivity in four out of 27 cases (15%) and >10 mm in 18 (40%) of cases with P53 positivity in 16 out of 18 cases (89%). The p-value of significance calculated was 0.00001. The tumor/stroma ratio interpreted by the criteria mentioned was low in 28 (62%) of the cases with P53 positivity in one out of 28 cases (4%) and high in 17 cases (38%) with P53 positivity in four out of 17 cases (24%). The p-value calculated was 0.11, with a chi-square value of 4, which was insignificant.

High Immune infiltration was seen in 38 cases (85%), with TP53 positivity in 16 out of 38 cases (42%), and low immune infiltration as per the criteria mentioned above was seen in seven cases (15%) with five of seven cases (71%) being P53 positive. The p-value calculated was 0.30, which was statistically insignificant. Tumor budding was scored as high, seen in 23 cases (51%), with TP53 positivity in 20 out of these 23 cases (87%), and low as per above mentioned criteria was seen in 22 cases (49%) with two out of 22 cases (9%) being P53 positive. The p-value calculated was 0.00001, which was statistically significant. The lymphovascular invasion was scored as absent in 41 cases (91%) with P53 positivity in one of 41 cases (2%) and present in four of the cases (9%) with P53 positivity in three of four cases (75%). The p-value of significance calculated was 0.0001. The perineural invasion was scored as absent in 40 cases (93%) with P53 positivity in one of 40 cases (3%) and present in five of the cases (7%) with P53 positivity in four of the five cases (80%). The p-value of significance calculated was 0.0003, which was statistically significant. The necrosis was scored as low in 40 cases (89%) with P53 positivity in one of 40 cases (3%) and present in five cases (11%) with P53 positivity in two out of five cases (40%). The p-value of significance calculated was 0.026, which was statistically insignificant. 

Based on the various histopathologic parameters, three groups were prepared (Table 5) with the highest frequency 23 out of 45 cases (51%) in the Histoscore group of 2-5.

**Table 5:** Histoscore groups for the different histoscores

Histoscores	Histoscore groups	Frequency of cases N(%)
Scores 2-5	1	23 (51%)
Scores 6-8	2	16 (36%)
Scores 9-11	3	6 (13%)

In oral squamous cell carcinoma (SCC), we observed significant associations between tumor grading, depth of invasion, tumor budding, lymphovascular invasion, perineural invasion, and immune cell infiltration at the invasion front, as confirmed by TP53 immunostaining. A p-value of 0.05 was regarded as statistically significant in our analysis. Therefore, these histopathological features, in conjunction with TNM staging, are valuable in predicting patient prognosis and should be carefully considered.

## Discussion

This retrospective observational study aimed to evaluate the histopathological parameters in SCC of oral cavity lesions in the biopsy specimens and establish its significance. The study evaluated each histopathology slide that featured SCC. Identifying malignant cases is crucial to detecting lesions early in the course of the disease for prompt treatment and improving prognosis. The clinical implications of the present study focuses and highlights the importance of reporting all the parameters of histopathology of SCC and sets a reminder for clinicians to always focus on all the pointers of a SCC report for prompt and early treatment.

In our present study, the age range of patients spanned from 24 to 95 years, which aligns with findings from various global studies. Our results also show that men had a higher incidence of oral mucosal lesions compared to women, consistent with reports by Dholakiya [17], Pudasaini and Barar [18], and Agrawal et al. [19]. Furthermore, the rising incidence of oral cancer is notably seen in individuals under 40 years, who make up an estimated 35% of all patients in our study. This aligns with findings by Swetha Acharya et al. [20], highlighting a concerning rise in disease occurrence among younger populations. Our study's higher proportion of younger patients can be largely attributed to early and frequent exposure to pan-tobacco chewing in this region. This early initiation of such habits may suggest a heightened vulnerability to the disease. Previous reports indicate that the rise of oral cancer in India is linked to the frequent use of pan masala products, which often contain tobacco and contribute to their carcinogenic effects [21].

In our study, the most common site affected was the buccal mucosa, followed by the tongue and then the lower lip. The majority of patients consumed tobacco in some form, which correlates with the fact that tobacco use is a known risk factor for developing oral cancer. The buccal mucosa is the most common site of involvement in malignant lesions 24 out of 45 cases (53%), similar to Modi et al. [22] and Wahi et al. [23], probably due to the use of tobacco. P53 expression was studied with respect to the histologic grade of malignancy. P53 positivity increased as the grade of malignancy increased. Since the oral cavity has abundant blood supply and lymphatic drainage, SCC has a very high chance of metastasis to the cervical region, which makes it a potentially fatal condition. Increasing histological severity has been linked to the buildup of P53 protein levels, according to Boyle et al. [24], Nylander et al. [25], Chiang et al. [26] and Watling et al. [27] who also investigated the positive correlation between P53 staining and rising grade of malignancy, similar to our study. A p-value of 0.003 was obtained. Past studies have suggested that nearly about half of the oral SCC cases contain P53 mutation [28].

Increased TP53 expression was seen in four of the four cases (100%) with lymphovascular invasion, which was in concordance with the study conducted by Giri et al. [29]. Immunopositivity with P53 was seen in three of three cases (100%) with perineural invasion similar to Kaur et al. [30].

The major limitation of our study was that it was a single institutional study making the sample size limited and availability of only one biomarker P53 for assessing various histopathological parameters.

## Conclusions

The oral cancer, particularly oral SCC, continues to pose a significant health threat, with increasing incidence rates, especially in Southeast Asia. The current TNM staging system, while widely used, does not fully capture the complexity of oral SCC's biological behavior, necessitating the integration of more reliable prognostic methods such as histopathological and molecular evaluations. This study highlights and focuses on the importance of always reporting the mentioned histopathological parameters, such as tumor-stroma ratio, immune response, and the presence of perineural and lymphovascular invasion, and not just a diagnosis in predicting patient outcomes. The study sets a alarm for the clinician to never avert these parameters mentioned in histopathology report, which could have a significant improvement in survival. The association between P53 expression and unfavorable histological features underscores the potential of this biomarker in improving prognostic accuracy. As research evolves, incorporating these advanced diagnostic methods could significantly enhance our ability to predict and manage oral cancer, ultimately leading to better patient outcomes and more effective treatments.
